# Anatomic description of the distal great saphenous vein to facilitate peripheral venous access during resuscitation: a cadaveric study

**DOI:** 10.1186/s13037-023-00351-2

**Published:** 2023-01-23

**Authors:** Samitha A. M. D. R. U. Senevirathne, Hesitha K. V. Nimana, Ratnasingam Pirannavan, Poorni Fernando, Karahin A. Salvin, Udari A. Liyanage, Ajith P. Malalasekera, Yasith Mathangasinghe, Dimonge J. Anthony

**Affiliations:** 1 Department of Anatomy, Genetics and Biomedical Informatics, Faculty of Medicine, University of Colombo, Colombo, Sri Lanka; 2 Department of Anatomy, Faculty of Medicine, University of Kelaniya, Kelaniya, Sri Lanka

**Keywords:** Great saphenous vein, Venous access, Cross-sectional anatomy, Venous cutdown, Cannulation, Anatomical landmarks

## Abstract

The distal great saphenous vein is a popular site for venous access by means of percutaneous cannulation or venous cutdown in a hemodynamically unstable patient. The aim of this study was to precisely define the surface anatomy and dimensions of the distal part of the great saphenous vein to facilitate the aforementioned procedures. Cross-sectional anatomy of the distal saphenous vein was studied in 24 cadaveric ankles sectioned at a horizontal plane across the most prominent points of the medial and lateral malleoli. The curvilinear distance from the most prominent point of the medial malleolus to the center of the saphenous vein, its widest collapsed diameter and skin depth were obtained. The great saphenous vein was located at a mean distance of 24.4 ± 7.9 mm anterior to the medial malleolus. The mean widest collapsed diameter was 3.8 ± 1.5 mm. The mean distance from the skin surface to the vein was 4.1 ± 1.2 mm. These measurements could be used to locate the saphenous vein accurately, particularly in hemodynamically unstable patients with visually indiscernible veins.

**Supplementary Information:**

The online version contains supplementary material available at 10.1186/s13037-023-00351-2.

## Introduction

The great saphenous vein is the longest vein in the body, which originates as a continuation of the medial marginal vein in the medial aspect of the dorsal venous arch of the foot [1]. It ascends anterior to the medial malleolus obliquely in the medial aspect of the leg and thigh and ends by draining into the femoral vein at the saphenofemoral junction after piercing the cribriform fascia [2].

Getting vascular access is of paramount importance for the resuscitation of an acutely ill patient [3]. Even though the upper limb veins are targeted first in gaining venous access, the distal great saphenous vein becomes a popular site in hemodynamically unstable patients with visually indiscernible veins, especially in a resource-poor setting [3]. Vascular access to the distal great saphenous vein can be achieved by percutaneous venous cannulation and distal saphenous venous cut-down, while the constant location of the vessel, its large caliber, having located on tough periosteum and thick wall of the vein facilitate these procedures [4].

The aim of this study was to describe the precise location of the distal great saphenous vein in relation to a prominent bony landmark and to obtain the external dimensions of the vessel which could help improve the success rate of percutaneous saphenous venous cannulation and saphenous venous cut-down procedure, especially in a resource-poor setting.

## Methods

We conducted a descriptive cross-sectional study on the self-donated cadavers at the Department of Anatomy, Genetics and Bioinformatics, Faculty of Medicine, University of Colombo. The study was approved by the institutional ethics review committee (EC/21/101). The required sample size was calculated according to Lwanga and Lemeshow [5] with a confidence level of 95% and an error of 0.21 using population estimates from a previous study [6].

We randomly selected 25 cadavers of Sri Lankan ethnicity. Cadavers with lower limb deformities, previous surgeries and fractures were excluded. The cadavers were fixed with phenoxyethanol as the main preservative as described elsewhere [7]. One lower limb per cadaver was sectioned at the knee joint plane and the calf and ankle circumferences were measured using a measuring tape as anthropometric parameters. The calf circumference was defined as the maximum girth of the calf as reported previously [8, 9]. The ankle circumference was measured at the level of the most prominent points of the medial and lateral malleoli. The lower limbs were then frozen at -20 ^0^C for 24 h and subsequently sectioned at a horizontal plane across the most prominent points of the medial and lateral malleoli, to study the surface anatomy and morphometry of the distal great saphenous vein. The specimens were stabilized and photographs were obtained using a high-definition digital camera from a fixed distance with a ruler placed adjacent to the specimen at the same plane.

**Fig. 1:**
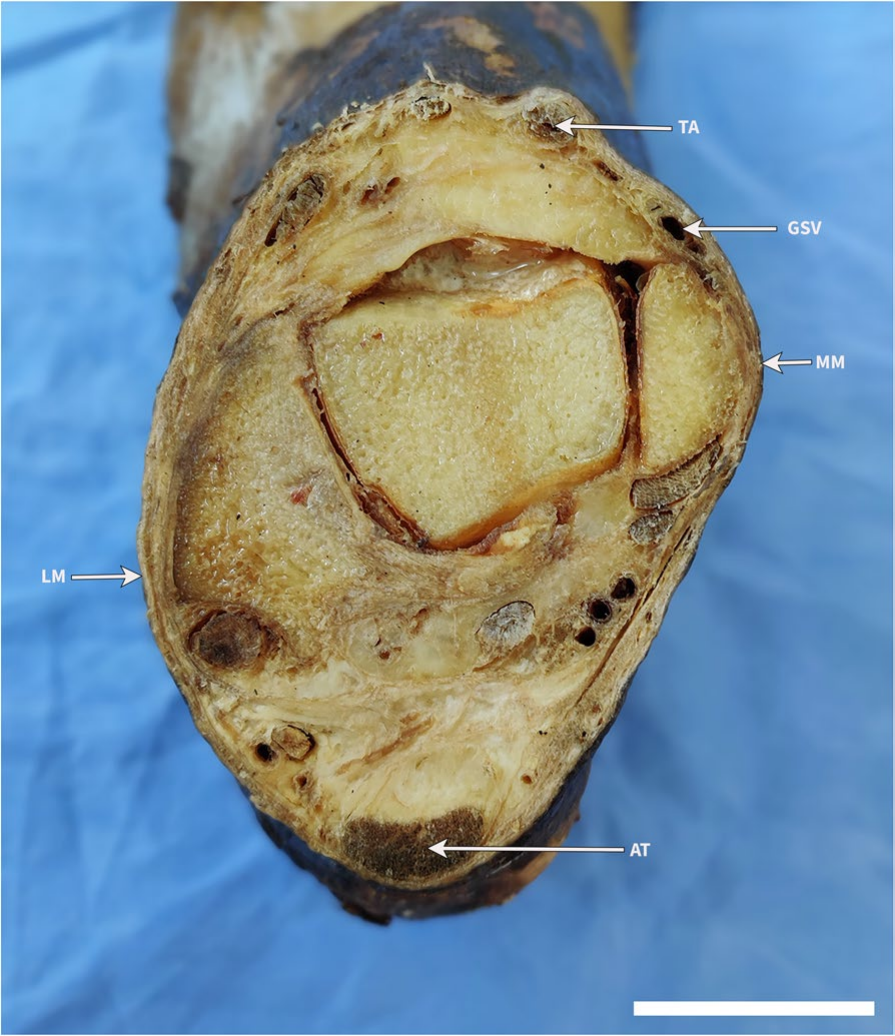
Cross-sectional anatomy at the horizontal plane across the most prominent bony points of medial and lateral malleoli. TA, Tibialis Anterior tendon. GSV, great saphenous vein. MM, the most prominent bony point of the medial malleolus. LM, the Most prominent bony point of the lateral malleolus. AT, Achilles tendon. Scale bar = 2 cm

**Fig. 2:**
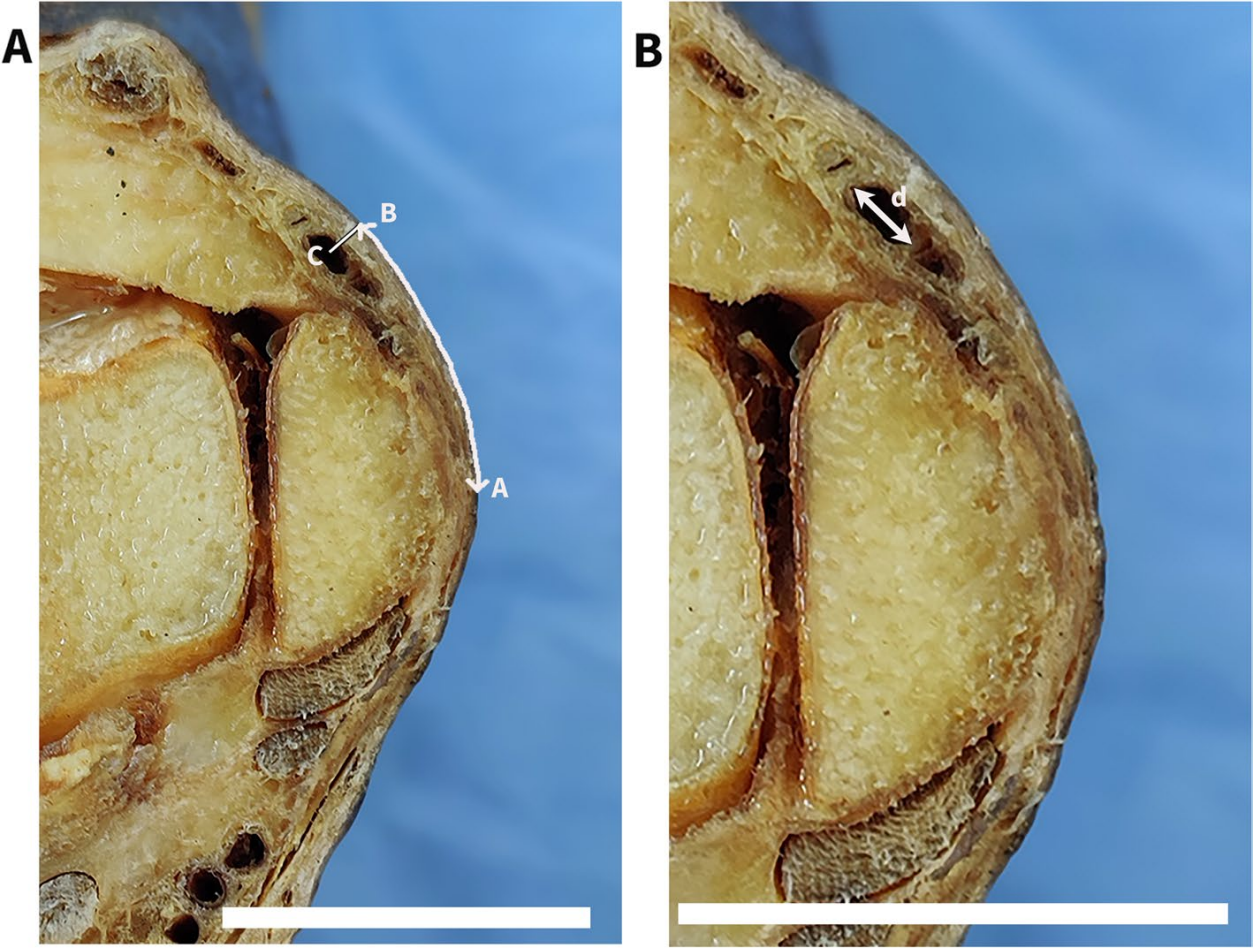
Measurements obtained in the study. A, most prominent bony point in medial malleolus. **B**, the point at which the perpendicular line drawn from the center of the great saphenous vein reaches the skin surface. **C**, the center of the great saphenous vein. **D**, widest collapsed diameter of the great saphenous vein. AB distance, curvilinear distance from the most prominent bony point of the medial malleolus to the great saphenous vein. BC distance, skin depth to the great saphenous vein. Scale bar = 2 cm

Photographs of the cross-sections were analyzed using Fiji (v1.53), an image processing software [10], using previously established methods [11, 12]. The curvilinear distance from the most prominent point of medial malleolus to the center of the saphenous vein, its widest collapsed diameter and skin depth were obtained (Figs. 1 and 2). In case of duplex systems, the measurements of the largest (dominant) vein were obtained. SPSS (v25.0) was used for the statistical analysis of data. Statistical significance was defined as *p* < 0.05. Data are reported as mean (standard deviation).

## Results

Twenty-five cadaveric ankles were initially included and one was later excluded due to distorted cross-sectional anatomy leaving 24 ankles (10 left, 14 right) for the study. The great saphenous vein was constantly located anterior to the medial malleolus in the superficial tissue plane in all the specimens. We found duplex great saphenous veins in three specimens (Supplementary Fig. 1). The great saphenous vein was located at a mean distance of 24.4 (SD 7.9) mm anterior to the medial malleolus. The mean widest collapsed diameter was 3.8 (SD 1.5) mm. The mean distance from the skin surface to the vein was 4.1 (SD 1.2) mm. Independent-sample t-test showed no statistically significant difference between the abovementioned measurements with the side of the ankle (*p* > 0.05).

The mean calf circumference was 26.3 (SD 2.6) cm. The calf circumference had a statistically significant negative correlation with the diameter of the saphenous vein (*p* = *0.045, r* = *-0.441*) indicating that the large calf circumferences were associated with smaller saphenous veins. Skin depth and the curvilinear distance from the medial malleolus to the great saphenous vein did not significantly correlate with calf circumference. In addition, the curvilinear distance from the medial malleolus to the great saphenous vein had a statistically significant positive correlation with the skin depth (*p* = *0.043, r* = *0.425*) depicting that the saphenous veins which are located more distant to the medial malleolus are located deeper in the superficial plane. The mean ankle circumference was 24.9 (SD 2.8) cm. The ankle circumference did not significantly correlate with the saphenous vein diameter, distance from the medial malleolus to the great saphenous vein or the skin depth.

## Discussion

This study demonstrates that the distal great saphenous vein was consistently located approximately 2.5 cm anterior to the medial malleolus, 4 mm deep to the skin and had a diameter of 4 mm. Previously reported distance between the medial malleolus and the vein is 2.5 cm [4], which agrees with our study. Similarly, the mean diameter of the vein is comparable to a previous study conducted on human saphenous venous grafts for cardiothoracic surgery where the reported diameter was 4.2 mm [6]. True duplications of the great saphenous vein at the ankle or accompanying large tributaries at this level, perhaps, could explain the double saphenous veins observed in our study [13, 14].

A multicentered randomized trial [15] reported that procedure duration for great saphenous venous cutdown (5.63 ± 2.58 min) was significantly higher compared to procedure duration for percutaneous femoral access (3.18 ± 1.19 min). Similarly, a Cochrane review [16] states that saphenous venous cutdown takes longer to carry out compared to intraosseous access. We believe that the deficiencies of knowledge about the surface anatomy and dimensions of the great saphenous vein could be one of the reasons for longer procedure times in obtaining great saphenous venous access. Therefore, using the dimensions described in our study may help improve the success rates of locating the saphenous vein, particularly in hemodynamically unstable patients with visually indiscernible veins in resource-poor settings.

### Shortcomings and limitations of the study

We only examined one lower limb per cadaver because the other limb was used for teaching purposes at our institute. Because of this limitation, we were unable to examine intra-individual lateral differences. Furthermore, we have not explored the sex differences. Larger studies may be necessary to ensure the generalizability of these results.

## Conclusions

The great saphenous vein was consistently located ~ 2.5 cm anterior to the medial malleolus and ~ 4 mm deep to the skin. These landmarks could be used to accurately locate the great saphenous vein in emergency procedures.

## Supplementary Information

**Additional file 1: Supplementary Figure 1. **Duplex great saphenous veins. Cross sections of the two veins are indicated by the arrows. Scale bar = 2 cm.

## Data Availability

The datasets used and/or analyzed during the current study are available from the corresponding author upon reasonable request.
